# Supplementary material for: Task-induced attention load guides and gates unconscious semantic interference
Source: Nat Commun. 2020 Apr 29;11:2088. doi: 10.1038/s41467-020-15439-x (PMC7190740; doi:10.1038/s41467-020-15439-x)
Supplement: Supplementary file 1 — Supplementary Information [file 41467_2020_15439_MOESM1_ESM.pdf]

Supplementary information

Task-induced attention load guides and gates unconscious semantic interference

Shao-Min Hung[1,3*], Daw-An Wu[1], Shinsuke Shimojo[1,2]

[1]Biology and Biological Engineering, California Institute of Technology, 1200 E. California Blvd., Pasadena, CA, 91125, USA
[2]Computation and Neural Systems, California Institute of Technology, 1200 E. California Blvd., Pasadena, CA, 91125, USA
[3]Huntington Medical Research Institutes, 686 South Fair Oaks Avenue, Pasadena, 91105, CA, USA

*Correspondence: smhung@caltech.edu

Supplementary Notes

1. Invisibility of the suppressed prime
2. Two-way ANOVA analyses on the raw reaction time in Experiments 3~6.
3. Negative priming in the Stroop paradigm
4. Low-level adaptation did not explain the unconscious interference

Supplementary Note 1

Invisibility of the suppressed prime. We ensured the invisibility of the suppressed time at two levels. Firstly, we looked at group-level performance. The interocular suppression was successful in making the prime invisible for the following reasons. First of all, participants' near-ceiling accuracy on the visible and blank catch trials showed that they were paying attention to the detection task under suppression (Supplementary Figure 1, Bottom). Secondly, during the suppression period, we also calibrated the prime luminance according to their detection on the suppressed prime with a staircase procedure. If some participant had been conservative and neglected primes that he/she has actually detected, we would expect the calibration to be a failure. Using a 3-up-1-down calibration staircase, the group average breakthrough rates on the suppressed prime in Experiments 1~8 were dawn in Supplementary Figure 1 (Top left) and shown in Table 1 in the main text. According to the 3-up-1-down staircase, we expected to see the final thresholds converge around 25% of the full contrast. These numbers suggest that the calibration was successful, and across participants there was little individual differences. Thirdly, their chance performance (i.e. 50%, above or below fixation) on the 2AFC prime location task objectively indicated the invisibility of the suppressed prime (Supplementary Figure 1, Top right).

Secondly, we examined in all experiments if each participant performed at chance level on the 2AFC prime location localization using a binomial test (one-tailed, $p > .05$) and re-analyzed the data with only those participants with chance performance[1]. In Experiment 1, 17 out of 20 participants performed at chance. we again performed three-way repeated measure ANOVA (*prime-target color consistency*, *prime-target word consistency*, and *target word-color consistency*). The main effects of target (reverse) Stroop, $F(1, 16) = 32.00$, $p = .00$, $\eta_p^2 = .67$. and color congruency, $F(1, 16) = 6.93$, $p = .02$, $\eta_p^2 = .30$ were found. Furthermore, there was a three-way interaction between color and word congruency as well as target Stroop, $F(1, 16) = 6.48$, $p = .02$, $\eta_p^2 = .29$. Post-hoc comparisons showed that double word-color incongruency significantly slowed down target response only when the target was not a Stroop word (paired $t(19) = -2.66$, $p = 0.02$, marginally significant after correction), but not when it was a Stroop word (paired $t(19) = -1.76$, $p = 0.10$). In Experiment 2, 16 participants were included, and the same analysis was

performed. The main effect of target Stroop was found, $F(1, 15) = 38.82$, $p = .00$, $\eta_p^2 = .72$, and no further effect from the suppressed prime was found (word: $F(1, 15) = 1.27$, $p = .28$, $\eta_p^2 = .08$; color: $F(1, 19) = 3.26$, $p = .09$, $\eta_p^2 = .18$). Identical analysis performed on the 1st Quarter trials showed very similar pattern to the overall data: The main effect of target Stroop was found, $F(1, 15) = 23.72$, $p = .0002$, $\eta_p^2 = .61$, with no further effect from the prime-target relationship (word: $F(1, 15) = 0.27$, $p = .61$, $\eta_p^2 = .02$; color: $F(1, 15) = 1.12$, $p = .31$, $\eta_p^2 = .07$). Similarly, the result on the 4th Quarter trials showed no main effects on both incongruency effect with word $F(1, 15) = 2.53$, $p = .13$, $\eta_p^2 = .14$ and color $F(1, 15) = 0.29$, $p = .60$, $\eta_p^2 = .02$, in addition to the effect of target Stroop, $F(1, 15) = 18.65$, $p = .0006$, $\eta_p^2 = .55$. These results are different from the data from twenty participants, which could be due to lack of statistical power. We will not draw any conclusion from these data. In Experiment 3, all participants performed at chance. In Experiment 4, 16 out of the 20 participants performed at chance. The word-induced incongruency effect was insignificant with $t(15) = -1.62$, $p = .13$, Cohen's $d_{av} = 0.52$, word congruent trials: 0.29 % faster; word incongruent trials: 5.95 % slower. However, with these 16 participants, the reaction time between 1stQ and 4thQ (1067 ms vs. 1045 ms) trials were not significant $t(15)= 0.84$, $p = 0.41$, (1067 ms vs. 1045 ms). Although this result appeared to support that without the practice effect, the task remained at the high load for these participants and the congruence effect thus remained inconsequential. However, an alternative explanation would be the lack of power as the two conditions still exhibited a difference of 6 % reaction time slowing. In Experiment 5, 19 out of 20 participants performed at chance. The color incongruency effect was not significant with $t(19) = 0.76$, $p = .45$, Cohen's $d_{av} = 0.24$, congruent trials: 5.47 % slower; incongruent trials: 3.82 % slower. In Experiment 6, 19 out of 20 participants performed at chance. The color incongruency effect remained insignificant, either in all trials ($t(19) = -0.26$, $p = .80$, Cohen's $d_{av} = 0.07$, congruent trials: 4.0 % slower; incongruent trials: 3.6 % slower) or later trials ($t(19) = 0.14$, $p = .89$, Cohen's $d_{av} = 0.04$, congruent trials: 4.81 % slower; semantic incongruent trials: 5.22 % slower). In Experiment 7, all participants performed at chance. In Experiment 8, 18 out of 20 participants performed at chance at the location task. The semantic incongruency effect again interacted with the location, showing the effect only when target and prime were co-localized. A direct comparison on the co-localized trials yielded similar significant results ($t(17) = -2.3$, $p = 0.03$, Cohen's $d_{av} = 0.63$, semantic congruent trials: 1.26 % slower; semantic incongruent trials: 5.71 % slower).

Furthermore, as we have used a floating thresholding procedure in all experiments, one may argue that different levels of subliminal prime luminance could occur and cause the differences across experiments. As Experiments 3, 4 and 7, 8 used the colorless (gray) primes and identical trial-by-trial thresholding, we looked into the breaking thresholds of these experiments to see if they were comparable. The average prime luminance levels in Experiments 3, 4, 7 and 8 were 10.56 (0.67) %, 9.39 (0.55) %, 11.12 (1.04) %, and 9.96 (0.80) %, with no difference between the four, $F(3, 76) = 0.90$, $p = 0.44$. However, we have to point out that in our study, different participants were recruited in different experiments, thus the luminance levels across experiments may not be a good indicator of suppression strength.

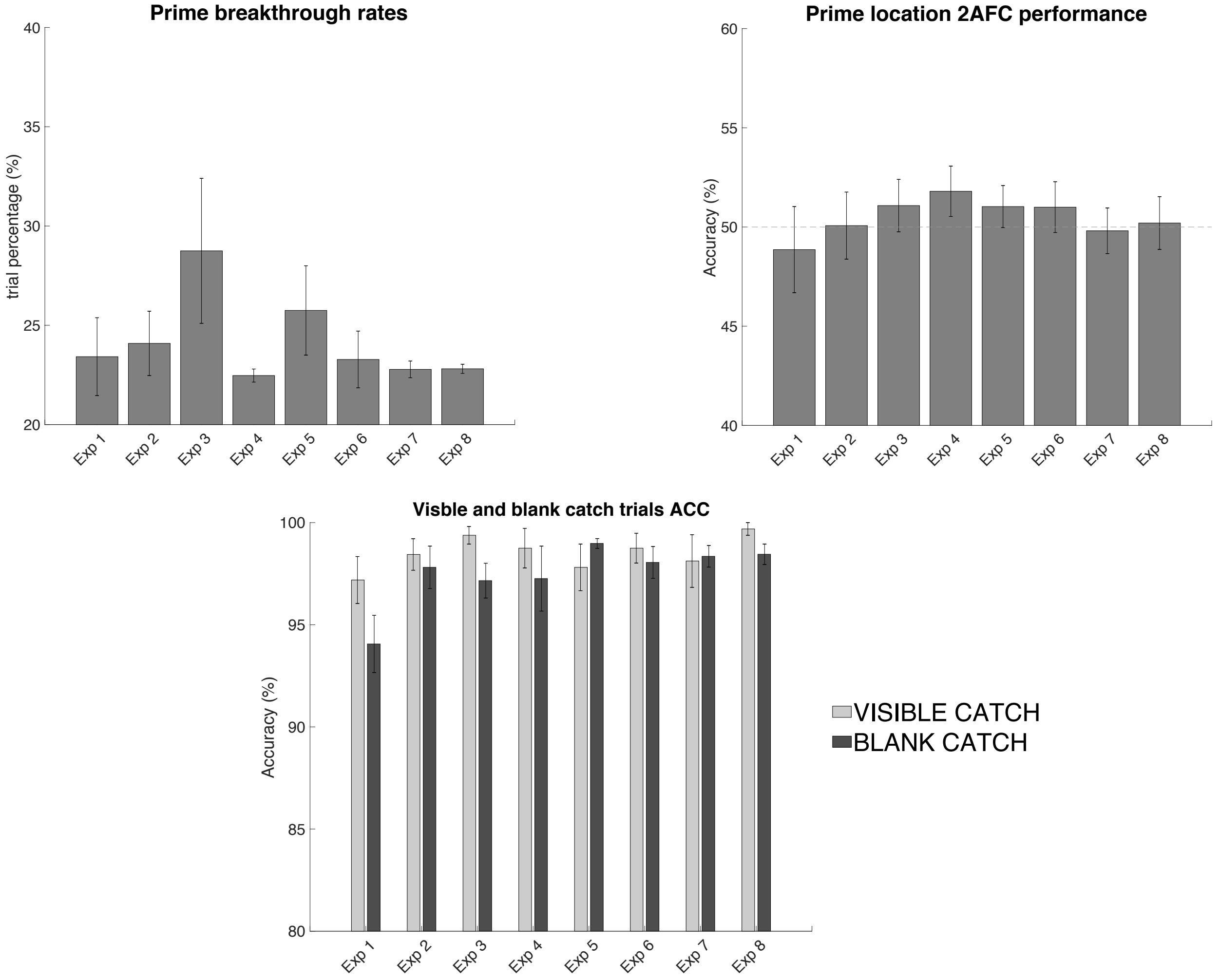

Supplementary Figure 1. **Top left.** The breakthrough rates of the suppressed prime in all experiments. **Top right.** 2AFC Prime location task accuracy in all experiments. **Bottom.** Accuracy on visible and blank catch trials in all experiments. Each bar represents group results (n=20) from each experiment. Error bars denote stand error of the mean (SEM).

Overall, our data showed that at the group level, participants paid attention to the detection task and responded accordingly, reflected by successful calibration results and near-ceiling performance on both blank and visible trials. Moreover, even when participants who performed above chance level on the 2AFC prime location task were excluded, the results showed similar patterns as we found in all participants. We agree that any of the single criterion was not sufficient to claim that participants were unconscious of the prime, and measuring stimulus visibility always has its unavoidable uncertainty. However, all above results elicited converging evidence of the prime invisibility.

Supplementary Note 2

Experiment 3. To examine if the congruency effect interacts with target reverse Stroop/non-Stroop, we also performed a two-way (word consistency; target (non)Stroop) repeated measures analysis of variance on the target word raw reaction time. We found significant main effects of target reverse Stroop/non-Stroop ($F(1, 19) = 9.25, p = .007, \eta_p^2 = .33$.) and prime-target word congruency ($F(1, 19) = 7.11, p = .02, \eta_p^2 = .27$.). The interaction between the two effects was not significant with $F(1, 19) = 0.09, p = .77, \eta_p^2 = .001$.

Experiment 4. Similarly, we also performed a two-way (word consistency; target (non)Stroop) repeated measures analysis of variance on the target word raw reaction time in all trials, 1st quarter trials, and 4th quarter trials. In all trials, we found significant main effect of target Stroop effect ($F(1, 19) = 19.83, p = .0003, \eta_p^2 = .51$.) but not prime-target word congruency ($F(1, 19) = 1.59$, $p = .22, \eta_p^2 = .08$.). The interaction between the two effects was not significant with $F(1, 19) = 1.78, p = .20, \eta_p^2 = .02$. Same analysis performed on 1st quarter trials showed similar results: significant main effect of target Stroop effect ($F(1, 19) = 13.62, p = .002, \eta_p^2 = .42$.) with no effect on prime-target word congruency ($F(1, 19) = 0.10, p = .75, \eta_p^2 = .01$.). The interaction between the two was not significant with $F(1, 19) = 0.07, p = .80, \eta_p^2 = .00$. On the contrary, in the 4th quarter trials, there were significant main effects of both target Stroop/non-Stroop ($F(1, 19) =$

7.68, $p = .01$, $\eta_p^2 = .29$.) and prime-target word congruency ($F(1, 19) = 4.17, p = .05, \eta_p^2 = .18$.) with no interaction between the two ($F(1, 19) = 0.21, p = .65, \eta_p^2 = .00$.

Experiment 5. To look into the interaction between prime-target congruency and target (non)Stroop, we also performed a two-way (color consistency; target (non)Stroop) repeated measures analysis of variance on the target word raw reaction time in Experiment 5. Similar to what we found with reaction percentage changes normalized with baseline blank trials, grouping trials according to the color-perceiving account showed only the main effect on the target Stroop effect ($F(1, 19) = 23.03, p = .0001, \eta_p^2 = .55$, with neither main effect on the color congruency ($F(1, 19) = 0.46, p = .51, \eta_p^2 = .02$, nor the interaction between the two ($F(1, 19) = 0.07, p = .80$, $\eta_p^2 = .001$).

Experiment 6. In Experiment 6, we also performed a two-way (color consistency; target (non)Stroop) repeated measures analysis of variance on the target word raw reaction time in all trials, 1st quarter trials, and 4th quarter trials. In all trials, we found significant main effect of target Stroop effect ($F(1, 19) = 37.37, p = .00, \eta_p^2 = .66$.) but not prime-target color congruency ($F(1, 19) = 0.93, p = .35, \eta_p^2 = .05$.). The interaction between the two effects was not significant with $F(1, 19) = 0.74, p = .40, \eta_p^2 = .01$. Same analysis performed on 1st quarter trials showed similar results: significant main effect of target Stroop effect ($F(1, 19) = 30.96, p = .00, \eta_p^2 = .62$.) with no effect on prime-target color congruency ($F(1, 19) = 0.01, p = .91, \eta_p^2 = .00$.). The interaction between the two was not significant with $F(1, 19) = 1.15, p = .30, \eta_p^2 = .02$. Similarly, in the 4th quarter trials, there was significant main effect on target Stroop/non-Stroop ($F(1, 19) = 15.97, p = .00, \eta_p^2 = .46$.) but not prime-target color congruency ($F(1, 19) = 2.75, p = .11, \eta_p^2 = .13$.) with no interaction between the two ($F(1, 19) = 0.02, p = .89, \eta_p^2 = .00$.

Supplementary Note 3

Negative priming in the Stroop paradigm. Our color-naming experiment (e.g. Experiment 2) showed that in the later trials when there was a significant practice effect on the target responses, incongruent prime-target words led to slower responses. This was different from the typical negative priming effect observed in the Stroop literature[2–4]. In the context of the Stroop paradigm, negative priming refers to the related relationship between a prime and target in an ignored stimulus dimension leads to interferences (e.g. slower responses). For example, Marí-Beffa et al.[4] examined that when the prime did not elicit the Stroop effect, was such null result sufficient to conclude that word processing did not occur, and thus word-reading was not automatic. They approached this question with a prime-target negative priming paradigm where after the prime, participants had to name of color of a target, which was always color-word inconsistent. They found that albeit no Stroop effect was found on the response to prime, there was a clear negative priming from the prime to the target. That is, when the two words were related, people responded *slower* to the target. This finding is different from the negative interference effect we report here in that we show that when the prime-target words were incongruent, people responded slower. One major difference is that in their design, the target was always color-word inconsistent. In order to correctly respond to the target, participants had to always suppress the word processing, and a related prime-target relationship made the suppression more difficult, leading to the negative priming. Such negative priming effect could indeed be suitable to be combined with our paradigm to further examine what exactly can be extracted and processed under interocular suppression.

Supplementary Note 4

Low-level adaptation did not explain the unconscious interference. Although in all experiments, the prime and target were of different font sizes and presented on jittered locations. We first proceeded to examine if prime-target co-localization (on the same or different sides of the fixation point) interacted with the word congruency effect with the reasoning that if stronger effect arose when the prime and target were spatially closer, low-level visual adaptation may contribute significantly to our word incongruency effect. We thus ran an additional two-way (word congruency; prime-target location consistency) repeated measures analysis of variance on the data of Experiments 3 & 4. The results from both experiments showed no interaction between location and semantic congruency (Experiment 3, all trials, $F(1, 19) = 0.41$, $p = 0.53$, $\eta_p^2 = .004$; Experiment 4, 4th quarter trials, $F(1, 19) = 2.12$, $p = 0.16$, $\eta_p^2 = .03$). Such results suggested that our findings that prime-target word incongruency slowed down target responses were not merely driven by low-level adaptation of visual stimulus shapes.

Supplementary References

1. Lin, Z. & Murray, S. O. Unconscious Processing of an Abstract Concept. *Psychological Science* **25**, 296–298 (2014).
2. Neill, W. T. Inhibitory and facilitatory processes in selective attention. *Journal of Experimental Psychology: Human Perception and Performance* **3**, 444–450 (1977).
3. Besner, D. The myth of ballistic processing: Evidence from Stroop's paradigm. *Psychonomic Bulletin & Review* **8**, 324–330 (2001).
4. Marí-beffa, P., Estévez, A. F. & Danziger, S. Stroop interference and negative priming: Problems with inferences from null results. *Psychonomic Bulletin & Review* **7**, 499–503 (2000).
