# Supplementary material for: Task-induced attention load guides and gates unconscious semantic interference
Source: Nat Commun. 2020 Apr 29;11:2088. doi: 10.1038/s41467-020-15439-x (PMC7190740; doi:10.1038/s41467-020-15439-x)
Supplement: Supplementary file 3 — Reporting Summary [file 41467_2020_15439_MOESM3_ESM.pdf]

nature research

Corresponding author(s): Shao-Min Hung

Last updated by author(s): Jan 24, 2020

Reporting Summary

Nature Research wishes to improve the reproducibility of the work that we publish. This form provides structure for consistency and transparency in reporting. For further information on Nature Research policies, see Authors & Referees and the Editorial Policy Checklist .

Statistics

For all statistical analyses, confirm that the following items are present in the figure legend, table legend, main text, or Methods section.

n/a	Confirmed	
☐	☒	The exact sample size (*n*) for each experimental group/condition, given as a discrete number and unit of measurement
☐	☒	A statement on whether measurements were taken from distinct samples or whether the same sample was measured repeatedly
☐	☒	The statistical test(s) used AND whether they are one- or two-sided<br>*Only common tests should be described solely by name; describe more complex techniques in the Methods section.*
☐	☒	A description of all covariates tested
☐	☒	A description of any assumptions or corrections, such as tests of normality and adjustment for multiple comparisons
☐	☒	A full description of the statistical parameters including central tendency (e.g. means) or other basic estimates (e.g. regression coefficient) AND variation (e.g. standard deviation) or associated estimates of uncertainty (e.g. confidence intervals)
☐	☒	For null hypothesis testing, the test statistic (e.g. *F*, *t*, *r*) with confidence intervals, effect sizes, degrees of freedom and *P* value noted<br>*Give P values as exact values whenever suitable.*
☒	☐	For Bayesian analysis, information on the choice of priors and Markov chain Monte Carlo settings
☐	☒	For hierarchical and complex designs, identification of the appropriate level for tests and full reporting of outcomes
☐	☒	Estimates of effect sizes (e.g. Cohen's *d*, Pearson's *r*), indicating how they were calculated

Our web collection on statistics for biologists contains articles on many of the points above.

Software and code

Policy information about availability of computer code

Data collection	MATLAB (Mathworks) 2014b; PsychToolBox 3.0.14
Data analysis	MATLAB (Mathworks) 2018a

For manuscripts utilizing custom algorithms or software that are central to the research but not yet described in published literature, software must be made available to editors/reviewers. We strongly encourage code deposition in a community repository (e.g. GitHub). See the Nature Research guidelines for submitting code & software for further information.

Data

Policy information about availability of data

All manuscripts must include a data availability statement. This statement should provide the following information, where applicable:
- Accession codes, unique identifiers, or web links for publicly available datasets
- A list of figures that have associated raw data
- A description of any restrictions on data availability

Data will be shared upon reasonable request. Individual data are drawn on the figures.

Field-specific reporting

Please select the one below that is the best fit for your research. If you are not sure, read the appropriate sections before making your selection.

☐ Life sciences ☒ Behavioural & social sciences ☐ Ecological, evolutionary & environmental sciences

For a reference copy of the document with all sections, see nature.com/documents/nr-reporting-summary-flat.pdf

Behavioural & social sciences study design

All studies must disclose on these points even when the disclosure is negative.

Study description	Quantitative experimental.
Research sample	Caltech community, including students, postdocs, and staffs.
Sampling strategy	Participants were recruited through the Caltech SONA system and internal mailing lists. A total number of 20 participants in each experiment was targeted based on our 80% power calculation in our previous study (Hung & Hsieh, 2015).<br><br>Hung, S. M. & Hsieh, P.-J. Syntactic processing in the absence of awareness and semantics. Journal of Experimental Psychology: Human Perception and Performance 41, 1376–1384 (2015).
Data collection	The data collection was performed with computerized MATLAB based script. Participants performed the tasks in a dark room by themselves and were all naive to the purpose of the experiment. The first author was the experimenter and was not blind to the purpose of the experiment.
Timing	2018 April - 2019 Sep
Data exclusions	Participants that (1) had performance 3 standard deviations away from the group mean on the catch tasks or/and (2) failed to achieve successful calibration on prime luminance or/and (3) brokethrough suppression yet failed to indicate prime location (see experimental design and procedure) were removed before entering analysis. (see Stimuli and Procedure of each experiment; Experiment 1: n = 3; Experiment 2: n = 2; Experiment 3: n = 1; Experiment 4: n = 2; Experiment 5: n = 0; Experiment 6: n = 0; Experiment 7: n = 1; Experiment 8: 2).
Non-participation	N/A
Randomization	Participants were not allocated into experimental groups.

Reporting for specific materials, systems and methods

We require information from authors about some types of materials, experimental systems and methods used in many studies. Here, indicate whether each material, system or method listed is relevant to your study. If you are not sure if a list item applies to your research, read the appropriate section before selecting a response.

Materials & experimental systems

n/a	Involved in the study	
☒	☐	Antibodies
☒	☐	Eukaryotic cell lines
☒	☐	Palaeontology
☒	☐	Animals and other organisms
☐	☒	Human research participants
☒	☐	Clinical data

Methods

n/a	Involved in the study	
☒	☐	ChIP-seq
☒	☐	Flow cytometry
☒	☐	MRI-based neuroimaging

Human research participants

Policy information about studies involving human research participants

Population characteristics	See above.
Recruitment	Participants were recruited through the Caltech SONA system and internal mailing lists.
Ethics oversight	Institutional Review Board, Caltech.

Note that full information on the approval of the study protocol must also be provided in the manuscript.
